# Setting the stage of innovations in allergy globally

**DOI:** 10.1186/1939-4551-7-5

**Published:** 2014-03-21

**Authors:** Alessandro Fiocchi

**Affiliations:** 1 Pediatric Hospital Bambino Gesù, Piazza di Sant’Onofrio 4, Rome, Vatican City, Italy

## 

This is my first editorial as the new Editor-in-Chief of the *World Allergy Organization Journal* (WAO Journal), and I must begin with a thank you to the previous Editor-in-Chief, Professor Lanny Rosenwasser. He had set for the Journal a high standard that I pledge to maintain and to develop further. During his tenure, the Journal was established as an open-access resource and became indexed in PubMed Central and PubMed as well as other open source indexes, and the number of articles increased significantly, making it a regular publication in its new online-only form. Both Professor Rosenwasser and our first Editor-in-Chief, Professor Johannes Ring, will thankfully stay involved as Founding Editors, so the Journal will have the opportunity to continue to profit from their combined experience.

In addition, I would like to reciprocate the wishes to Professor Rosenwasser [1] for a fruitful presidency. In the next two years he will lead an organization that has measurably expanded. What was done during the last presidencies to recruit excellent resources for the World Allergy Organization (WAO) is an unplleled effort among the medical scientific societies. The team built under the presidency of Professor G. Walter Canonica, with the formation of the Special Committees, paved the way to the creation of authoritative guidelines in his time and throughout the presidencies of Professor Richard F. Lockey and Professor Ruby Pawankar. This made the organization an influential collaborator with patient organizations, regulatory authorities, and industry everywhere. During the past two years, WAO established several scientific initiatives and position papers focusing on severe and complex allergies, [2] nurtured connections with a number of national and regional allergy societies, in particular in Asia and Africa, organized high caliber scientific meetings, and promoted initiatives both in countries where allergic disease is a big health problem and in countries where it is growing in prevalence and severity [3]. This was due to the dedicated efforts of a wise leadership led by Professor Ruby Pawankar. This gave me personally the opportunity to lead an important initiative, the development of Guidelines on Atopic Disease Prevention (GLAD-P), in line with her vision which included a focus on disease prevention.

I have no doubt that the new presidency will further extend the WAO horizons. Thus, while Professor Rosenwasser is receiving a growing organization, I am taking the leadership of a very interesting and growing Journal, now entering its seventh year of publication, with a substantial number of articles covering the main topics of our discipline, i.e., (in decreasing order of citations) asthma, allergic rhinitis, food allergy, immunotherapy, and drug allergy. Most important, the *WAO Journal* is ready for citation analysis toward acquiring its Impact Factor.

Being the official organ of the World Allergy Organization, WAO Journal is intended to mirror the professional and academic interests of our members. It will also be a platform for viewing the development of allergic disease in the world. As a pediatric allergist active in the last 25 years, I have experienced the clinical impact of the increase of this new class of diseases: increasing number of patients, increasing complexity of cases, and new allergic pathologies. To exemplify, our generation witnessed the appearance of the Oral Allergy Syndrome [4], the Food Protein-Induced Enterocolitis Syndrome, [5] and Eosinophilic Oesophagitis [6]. As a reader of the allergy literature, I would have hardly imagined 25 years ago that in 2012 we would have 23 indexed allergy journals, with appreciable to high ranking impact factors.

The *WAO Journal* will profit from the tremendous advancements reflected in these journals and will attempt to keep the reader updated on the different aspects of allergy diagnosis, interpretation, and treatment. For diagnosis and treatment, in particular, the availability of molecular tools opens new avenues to a better interpretation of the disease [7], the formulation of new forms of specific immunotherapy [8], and the generation of molecular treatments able to target very specific aspects of allergic disease [9]. Specifically, the new Deputy Editor of *WAO Journal*, Professor Erika Jensen-Jarolim, as an expert in the field has followed the progress of diagnostics and molecular therapy since the cloning of the first allergen to the borders with oncology [10]. She will orient the Journal in the field of clinical interpretation of molecular-based diagnostics. This is one of the main interests of WAO, as focused on in a recent consensus document [11]. WAO is assembling a strong Editorial Board which Erika and I are proud of. It will profit from the experience of young researchers as well as of prominent scientists and former presidents of the organization. One of them, Professor Michael Kaliner (President, 2005–2007), will be Series Editor for the new section he initiated and launched, “WAO International Case-based Discussions”, [12] aimed to illuminate the different perspectives of the same clinical problem from the different parts of the world.

The causes of allergy epidemics are far from being fully understood and remain a real mystery of modern life [13]. I am sure that research on the etiology and mechanisms of allergic disease (s) will be one of the major topics of *WAO Journal* over the next few years. In this respect, we have still to learn from epidemiologic studies that are being actively developed. The comparison among the allergy trends in the different parts of the world will be particularly interesting in this regard, and the Journal is the natural place for comptive epidemiologic studies [14,15].

All active individual members of the 92 WAO Member Societies are members of WAO. This Journal is their publication. It is your publication. Thus, I encourage you to participate in the *WAO Journal* endeavor to publish works with specific clinical relevance and practical measures that are critical for the practice of allergy anywhere in the world.
